# Effect of flash glucose monitoring in adults with type 1 diabetes: a nationwide, longitudinal observational study of 14,372 flash users compared with 7691 glucose sensor naive controls

**DOI:** 10.1007/s00125-021-05437-z

**Published:** 2021-03-27

**Authors:** David Nathanson, Ann-Marie Svensson, Mervete Miftaraj, Stefan Franzén, Jan Bolinder, Katarina Eeg-Olofsson

**Affiliations:** 1 Department of Medicine, Karolinska University Hospital Huddinge, Karolinska Institute, Stockholm, Sweden; 2 Department of Molecular and Clinical Medicine, Sahlgrenska Academy, University of Gothenburg, Gothenburg, Sweden; 3 Centre of Registers Västra Götaland, Gothenburg, Sweden; 4 Health Metrics, Department of Public Health and Community Medicine, Sahlgrenska Academy, University of Gothenburg, Gothenburg, Sweden; 5 Department of Medicine, Sahlgrenska University Hospital, University of Gothenburg, Gothenberg, Sweden

**Keywords:** Continuous glucose monitoring, Flash glucose monitoring, Glucose control, Hypoglycaemia, Type 1 diabetes

## Abstract

**Aims/hypothesis:**

The aim of this work was to evaluate changes in glycaemic control (HbA_1c_) and rates of severe hypoglycaemia over a 2 year period after initiation of flash glucose monitoring (FM) in type 1 diabetes.

**Methods:**

Using data from the Swedish National Diabetes Registry, 14,372 adults with type 1 diabetes with a new registration of FM during 2016–2017 and with continued FM for two consecutive years thereafter, and 7691 control individuals using conventional self-monitoring of blood glucose (SMBG) during the same observation period, were included in a cohort study. Propensity sores and inverse probability of treatment weighting (IPTW) were used to balance FM users with SMBG users. Changes in HbA_1c_ and events of severe hypoglycaemia were compared.

**Results:**

After the start of FM, the difference in IPTW change in HbA_1c_ was slightly greater in FM users compared with the control group during the follow-up period, with an estimated mean absolute difference of −1.2 mmol/mol (−0.11%) (95% CI −1.64 [−0.15], −0.75 [−0.07]; *p* < 0.0001) after 15–24 months. The change in HbA_1c_ was greatest in those with baseline HbA_1c_ ≥70 mmol/mol (8.5%), with the estimated mean absolute difference being −2.5 mmol/mol (−0.23%) (95% CI −3.84 [−0.35], −1.18 [−0.11]; *p* = 0.0002) 15–24 months post index. The change was also significant in the subgroups with initial HbA_1c_ ≤52 mmol/mol (6.9%) and 53–69 mmol/mol (7.0–8.5%). Risk of severe hypoglycaemic episodes was reduced by 21% for FM users compared with control individuals using SMBG (OR 0.79 [95% CI 0.69, 0.91]; *p* = 0.0014)].

**Conclusions/interpretation:**

In this large cohort, the use of FM was associated with a small and sustained improvement in HbA_1c_, most evident in those with higher baseline HbA_1c_ levels. In addition, FM users experienced lower rates of severe hypoglycaemic events compared with control individuals using SMBG for self-management of glucose control.

**Graphical abstract:**

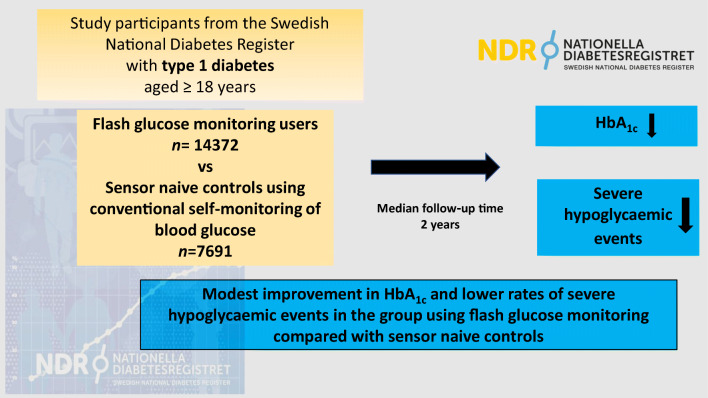

**Supplementary Information:**

The online version contains peer-reviewed but unedited supplementary material available at 10.1007/s00125-021-05437-z.

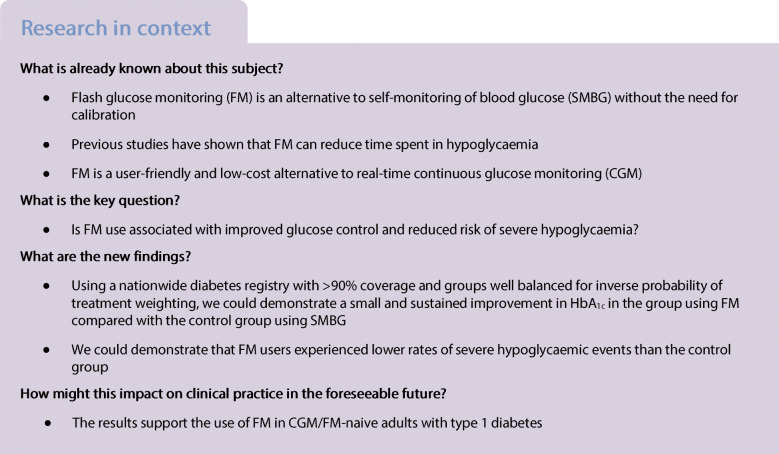



## Introduction

The benefit of optimal glucose control in reducing the risk for diabetes-related complications is an accepted paradigm. To attain glycaemic targets, glucose sensor-based technologies are probably the most innovative recent clinical advancements to aid self-management of glycaemic control. As stand-alone devices, systems for real-time continuous glucose monitoring (CGM) enable the user to gain instant information on the current glucose control and temporal trend, include alarms for hypo- and hyperglycaemia, and permit detailed retrospective analyses of day and night glucose control. In more complex integrated systems with insulin pumps the administration of insulin may be regulated based on sensor glucose readings.

In 2014, another technology, based on intermittently scanned glucose measurements (also named flash glucose monitoring [FM]), became available. Unlike CGM, this device requires active self-scanning of the glucose sensor and may therefore be viewed as a more convenient alternative to conventional self-monitoring of blood glucose (SMBG). The sensor is factory-calibrated without the need for manual calibration during the 14 days wear time, and the accuracy is comparable with other CGM systems [1, 2].

CGM and FM are increasingly used in the management of type 1 diabetes [3, 4]. In Sweden, real-time CGM and FM have been reimbursed since 2014. This initially applied to selected people with type 1 diabetes on multiple daily insulin injections or insulin pump therapies with grossly inadequate glycaemic control and subsequently with wider indications, in particular, recurrent hypoglycaemia. Registration of CGM or FM use has been entered into the Swedish National Diabetes Registry (NDR) since 2016. Currently, 70% of all adults with type 1 diabetes in Sweden use these technologies, albeit with regional differences ranging between 33% and 82%, and with FM being the predominantly prescribed device [5].

Several observational studies have demonstrated improved glucose control, less hypoglycaemia and improved quality of life using FM [6, 7]. However, apart from the IMPACT studies, which showed reduced exposure to hypoglycaemia without worsening in HbA_1c_ in adults with well-controlled type 1 diabetes [8, 9], there are no randomised controlled trials or large longer-term population-based observational studies with well-matched non-FM users that have evaluated the efficacy of FM in type 1 diabetes.

In the present study, we have identified all adults with type 1 diabetes in the NDR who have initiated and maintained FM use for two consecutive years and those who have remained CGM/FM naive during the same time period, to allow propensity-score-adjusted analyses of long-term changes in glucose control after initiation of FM in comparison with conventional SMBG. In addition, in this nationwide survey we assessed the efficacy of FM in alleviating the incidence of severe hypoglycaemic events.

## Methods

### Data sources

The Swedish NDR is a nationwide registry with >90% coverage that has been described previously [10, 11]. The NDR was initiated in 1996 with annual reporting of clinical data, laboratory values, treatments and examinations by trained physicians and nurses. Most individuals with type 1 diabetes attend specialised outpatient clinics, and the type 1 diabetes diagnosis is based on clinician diagnosis including measures of autoantibodies. Reporting of CGM and FM use to the NDR has been made since 2016.

### Study population

Adults with type 1 diabetes treated with either multiple daily injections (MDI) of insulin or continuous subcutaneous insulin infusion (CSII), with a diabetes duration ≥1 year, with no registration of CGM or FM use in the NDR prior to the index date and with registration of FM use for at least two consecutive years during the study period from 2016 to 2018 were included in the FM treated cohort. Control individuals without any registration of FM or CGM use during the same period were also identified through the NDR using an identical method.

### Outcomes

HbA_1c_ data were retrieved from the NDR 3 years before and two consecutive years after initiation of FM; the index date for initiation of FM was defined as the first registration of FM use in the NDR.

Events of severe hypoglycaemia requiring third-party assistance were also obtained from the NDR (reported annually from no event up to more than five events per year).

### Statistical analyses

Descriptive statistics are presented in terms of averages with SD for continuous variables and count with percentage for discrete variables. Generalised additive modelling [12] was used to visualise HbA_1c_ before and after start of FM as smooth function of time. The main analysis was adjusted for confounding using inverse probability of treatment weighting (IPTW) based on propensity scores [13, 14], defined as the probability of being exposed to FM given the observed confounders. The idea behind IPTW is that each person is fitted with an analysis weight, where the weights are defined as ‘1/propensity score’ for the FM users and ‘1/(1 − propensity score)’ for the CGM/FM naive control individuals. This way individuals with a specific set of characteristics (i.e. a specific value of the propensity score) are upweighted to represent the number of individuals with those characteristics in the whole dataset regardless of treatment group, making a weighted comparison balanced with respect to all confounders contributing to the propensity score. The advantage of IPTW over propensity score matching is that none of the data are discarded and that we are able to estimate the average treatment effect for everyone (ATE), not achievable using the standard greed 1–1 propensity score matching. The way we define the analysis weights leads to an estimate of the ATE, which corresponds to everyone using an FM device compared with no-one using the device. The balance prior to and after applying the weights were investigated using the standardised mean difference (SMD) [15], where an SMD <0.2 was taken to indicate adequate balance and an SMD <0.1 to indicate excellent balance. The propensity scores were estimated using a machine learning algorithm, in this case a generalised boosted regression model (GBM) [16], which, just as a logistic regression model would, models the binary indicator of FM use as a function of a range of potential confounders but without the assumption of a linear association between the confounders and the log odds of CGM usage. The GBM includes pre-index observations on age, sex, diabetes duration, use of insulin pump, HbA_1c_, BMI, systolic and diastolic BP, total cholesterol, LDL- and HDL-cholesterol, triacylglycerols, kidney function (eGFR), albuminuria, smoking, physical activity, retinopathy, events of severe hypoglycaemia, ischaemic heart disease and stroke, with an interaction depth of 3, a maximum of 20,000 trees and a shrinkage of 0.005. The optimal number of trees was selected using a stopping rule minimising to the degree of imbalance as measured by the average weighted SMD across all confounders. The optimal number of trees was 11,226. The change in HbA_1c_ was then compared between FM users and CGM/FM naive control individuals using a weighted ANCOVA with robust SEs. Analyses of severe hypoglycaemia included crude proportions, and ITPW adjusted logistic regression analysis, again with robust SEs, of individuals with at least one post-index severe hypoglycaemic event, comparing FM users with control individuals. Missing values were imputed using multiple chained equations creating ten imputed data sets. The propensity scores were estimated separately for each imputed dataset and then averaged before turning them into IPTW analysis weights. The unadjusted analysis in categorised HbA_1c_ groups was performed for individuals with non-missing pre-index HbA_1c_ values (11,004 FM users and 5383 control individuals).

The analysis was done using R 11.0 (Foundation for Statistical Computing, Vienna, Austria; https://www.R-project.org/).

### Patient and public involvement

The study did not conduct any interaction or intervention with patients included in the registry. Ethical approval has been granted by the Regional Ethics Review Board at the University of Gothenburg, and all patients have given informed consent for participating in the NDR. Patients or public were not involved in the design, analysis or interpretation of the data. All analysed data were anonymised. Thus, it is not possible to track any of our aggregated variables to specific individuals.

## Results

### Participant characteristics

**Fig. 1:**
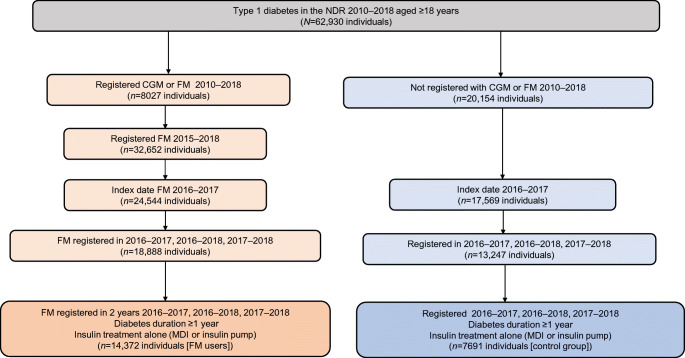
Flow chart for selection process of the study population

A detailed flow chart of the selection process of the study cohorts is presented in Fig. 1. In total 14,372 individuals with a registration of FM use and 7691 non-CGM/FM users were included. Median (IQR) follow-up time was 2.0 (1.6–2.3) years. Baseline characteristics of both study groups are shown in Table 1. For FM users, the mean (SD) age was 45 (17) years, 45% were of male sex, mean (SD) diabetes duration was 24 (14.9) years, mean (SD) BMI was 26 (4) kg/m^2^, 21% were insulin pump users, 67% had retinopathy and 10% were smokers. The corresponding data for the control group were mean (SD) age 55 (18) years, 41% of male sex, mean (SD) diabetes duration 26 (17) years, mean (SD) BMI 26 (4) kg/m^2^, 9% insulin pump users, 64% with retinopathy and 12% smokers. After IPTW, the groups of FM users and the CGM/FM naive controls using SMBG were well balanced with SMD <5% (Table 1).

**Table 1:** Baseline characteristics for control individuals and FM users with crude descriptive statistics and descriptive statistics after balancing the groups with the IPTW

Characteristic	Crude descriptive statistics			Descriptive statistics after balancing^a^		
	Control group (*N* = 7691)	FM users (*N* = 14,372)	SMD^b^	Control group	FM users	SMD^c^
Age, years (SD)	54.71 (18.3)	45.10 (16.5)	0.55	48.75 (17.82)	47.88 (17.51)	0.049
Male sex, *n* (%)	3130 (40.7)	6494 (45.2)	0.09	(42.7)	(43.7)	0.020
Diabetes duration, years (SD)	26.01 (17.1)	23.70 (14.94)	0.14	24.65 (15.79)	24.47 (15.64)	0.012
Insulin pump users (CSII), *n* (%)	654 (8.5)	3065 (21.3)	0.37	(16.1)	(17.4)	0.035
HbA_1c_, mmol/mol (SD)	60.96 (12.95)	63.38 (13.29)	0.184	62.21 (13.06)	62.64 (13.10)	
HbA_1c_, % NGSP (SD)	7.7 (1.2)	7.9 (1.2)		7.8 (1.2)	7.9 (1.2)	0.033
BMI, kg/m^2^ (SD)	25.95 (4.39)	25.96 (4.27)	0.002	25.90 (4.25)	25.91 (4.25)	0.003
Systolic BP, mmHg (SD)	130.00 (15.26)	126.54 (14.36)	0.233	127.79 (14.67)	127.58 (14.63)	0.015
Diastolic BP, mmHg (SD)	73.22 (9.44)	73.88 (9.12)	0.071	73.67 (9.10)	73.71 (9.19)	0.004
LDL-cholesterol, mmol/l (SD)	2.45 (0.84)	2.48 (0.81)	0.045	2.47 (0.81)	2.47 (0.80)	0.002
HDL-cholesterol, mmol/l (SD)	1.65 (0.52)	1.66 (0.50)	0.025	1.66 (0.50)	1.66 (0.51)	0.001
Total cholesterol, mmol/l (SD)	4.57 (0.99)	4.60 (0.94)	0.028	4.59 (0.95)	4.59 (0.95)	0.002
Triacylglycerols, mmol/l (SD)	1.13 (0.74)	1.05 (0.69)	0.115	1.07 (0.69)	1.07 (0.69)	0.008
Creatinine, μmol/l (SD)	81.45 (46.17)	76.87 (38.45)	0.108	78.17 (38.34)	77.87 (39.21)	0.008
eGFR^d^, ml min^−1^ [1.73 m]^−2^ (SD)	87.19 (26.78)	92.90 (25.13)	0.220	90.90 (25.65)	91.20 (25.41)	0.012
Albuminuria, *n* (%)			0.095			0.010
No albuminuria	5308 (82.0)	10,520 (85.3)		(84.8)	(84.5)	
Previous albuminuria	202 (3.1)	377 (3.1)		(2.9)	(3.0)	
Microalbuminuria	701 (10.8)	1033 (8.4)		(8.9)	(9.0)	
Macro albuminuria	262 (4.0)	410 (3.3)		(3.4)	(3.5)	
Physical activity, *n* (%)			0.183			0.024
Never	621 (9.8)	783 (6.4)		(7.6)	(7.1)	
< Once weekly	869 (13.8)	1600 (13.2)		(13.4)	(13.4)	
Once or twice weekly	1288 (20.4)	2802 (23.1)		(21.8)	(22.3)	
Three to five times weekly	1683 (26.7)	3892 (32.0)		(30.2)	(30.6)	
Daily activity	1850 (29.3)	3075 (25.3)		(27.0)	(26.6)	
SH, *n* (%)			0.031			0.020
No SH	6304 (94.7)	12,122 (94.2)		(94.8)	(94.4)	
One or two SH episodes registered	282 (4.2)	625 (4.9)		(4.2)	(4.6)	
Three to five SH episodes registered	42 (0.6)	72 (0.6)		(0.5)	(0.6)	
> Five SH episodes registered	27 (0.4)	48 (0.4)		(0.4)	(0.4)	
Ischaemic heart disease, *n* (%)	720 (10.1)	810 (6.0)	0.149	(7.1)	(7.1)	0.001
Retinopathy, *n* (%)	4513 (64.4)	9032 (66.9)	0.052	(65.6)	(66.3)	0.015
Stroke, *n* (%)	374 (5.3)	370 (2.8)	0.128	(3.7)	(3.4)	0.018
Smoker, *n* (%)	801 (11.6)	1387 (10.4)	0.037	(10.8)	(10.6)	0.006

Data are presented as *n* (%) for categorical variables or mean (SD) for continuous ariables. With the exception of age, male sex and diabetes duration all variables are subject to missing data. The absolute and relative frequencies are derived from the persons with non-missing information for each variable in each treatment group, as are the averages and standard deviations

^a^*n* is not shown after weighting

^b^SMD before weighting

^c^SMD after weighting

^d^eGFR calculated with the MDRD formula

NGSP, National Glycohemoglobin Standardization Program; SH, severe hypoglycaemia

### HbA_1c_

**Fig. 2:**
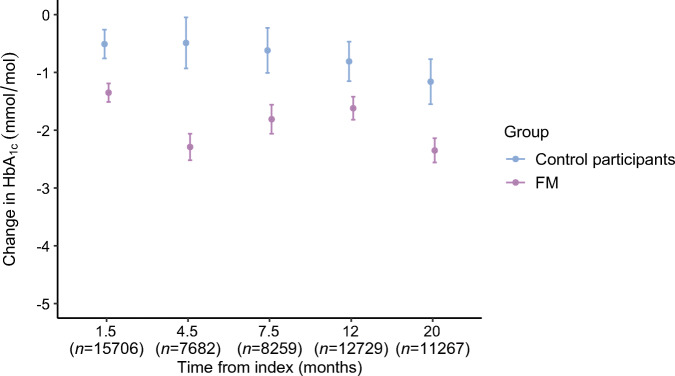
Weighted (IPTW) change in HbA_1c_ and 95% CIs for FM users and control individuals during the observational period from index up to 20 months after index. *n*=the total number of observations contributing at each time point

**Fig. 3:**
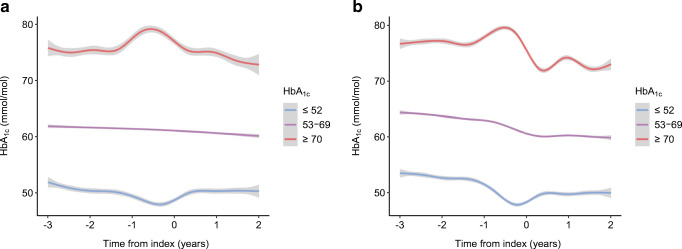
General updated mean HbA_1c_ for FM users (**a**) and for control individuals (**b**), categorised into low (≤52 mmol/mol), intermediate (53–69 mmol/mol) and high (≥70 mmol/mol) HbA_1c_ levels. The graphs show unadjusted data, therefore a comparison between the groups based on the figure should be made cautiously as data are not adjusted for confounders. The shaded area depicts indicative 95% CIs for the smooth functions of HbA_1c_. Note that the 95% CI does not account for multiple repeated measures from the same individual. The spikes in the graph depicting general updated mean for HbA_1c_ observed pre-index in the group with HbA_1c_ ≥70 mmol/mol and the pre-index dips in the group with HbA_1c_ ≤52 mmol/mol are caused by the effect of the ‘regression to the mean’ and are accordingly not associated with an effect of an intervention or selection bias

The changes (ITPW) in HbA_1c_ over the post-index 2 year observation period for FM users and CGM/FM naive controls is depicted in Fig. 2. In both groups, HbA_1c_ decreased gradually over time but the difference in weighted change in HbA_1c_ was significantly greater (*p* < 0.001) in the FM group compared with the control group at all time intervals after initiation of FM, with an estimated mean absolute difference of −1.2 mmol/mol (−0.11%) (95% CI –1.64 [−0.15], −0.75 [−0.07]; *p* < 0.0001) after 15–24 months. We also categorised FM users and control individuals according to baseline HbA_1c_ levels into three clinically relevant subgroups based on pre-index values: ≤52 mmol/mol (6.9%) (guideline target level); 53–69 mmol/mol (7.0–8.5%) (intermediate level); and ≥70 mmol/mol (8.6%) (inadequate diabetes control). The baseline characteristics of all subgroups are given in electronic supplementary material (ESM) Tables 1, 2. Mean, unadjusted change in HbA_1c_ was most marked in the highest HbA_1c_ subgroup (reduction of 8.5 mmol/mol [0.78%] at 15–24 months after starting FM), whereas the changes were less apparent in the two subgroups with lower basal HbA_1c_ levels (Fig. 3a). The corresponding descriptive analysis of HbA_1c_-divided subgroups among the control individuals are shown in Fig. 3b. The weighted (IPTW) difference in HbA_1c_ between FM users and CGM/FM naive control individuals was greatest in the subgroup with basal HbA_1c_ levels ≥70 mmol/mol (8.6%); the estimated mean absolute difference was −2.5 mmol/mol (−0.23%) (95% CI −3.84 [−0.35], −1.18 [−0.11]; *p* = 0.0002) 15–24 months post-index. The corresponding IPTW differences between FM users and control individuals in the intermediate (53–69 mmol/mol [7.0–8.5%]) and optimum (52 mmol/mol [6.9%] and below) HbA_1c_ subgroups were smaller, with estimated mean differences of −0.7 mmol/mol (−0.07%) (95%CI −1.1 [−0.10], −0.2 [−0.02]; *p* < 0.01) and −1.3 mmol/mol (−0.12) (95% CI −2.0 [−0.18], −0.5 [−0.05]; *p* < 0.001), respectively. Descriptive analysis showed similar findings when the HbA_1c_ subgroups were categorised according to sex (ESM Fig. 1, 2).

We examined the goodness of fit for the models. The variability greatly exceeded the signal producing R-square generally between 1 and 4%. Specifically, the R-square numbers were 1%, 0.4% and 2% for the control group, ranging from the lowest HbA_1c_ group to the highest. The corresponding values for the FM users were all 3%.

### Severe hypoglycaemia

Least-squares mean estimates of the proportion of individuals with at least one post-index event of severe hypoglycaemia were 4.1% (95% CI 3.8, 4.5) in the FM group and 5.2% (95% CI 4.7, 5.8) in the CGM/FM naive control group, respectively. The likelihood of experiencing one or more severe hypoglycaemic episodes was reduced by 21% for FM users compared with controls using SMBG (OR 0.79 [95% CI 0.69, 0.91]; *p* = 0.0014).

## Discussion

In this nationwide, longitudinal observational study, we examined the long-term effectiveness of FM on glucose control in adults with type 1 diabetes. Comparison was made with FM/CGM-naive control individuals, using propensity-score-adjusted analyses. We found that HbA_1c_ was marginally, albeit significantly, lower in the FM user group compared with the control group throughout the entire 2 year observational period, with the largest reduction in absolute terms after initiation of FM observed in those with the highest initial HbA_1c_ levels. We also showed that the FM users had a 21% lower risk of experiencing one or more severe hypoglycaemic events compared with the control group using conventional SMBG for self-management of glucose control.

While FM is being increasingly used as a replacement for SMBG, the current evidence on the effect of FM on HbA_1c_ in type 1 diabetes is limited and largely confined to observational data. In a prospective, observational study by Tyndall and colleagues [17], including 900 adults with type 1 diabetes starting FM use and a non-matched comparator group of 518 individuals with no FM, the median change in HbA_1c_ was −4 mmol/mol (−0.3%) in favour of the FM users after a median follow-up period of 245 days [17]. In a recent multicentre survey from Belgium involving almost 2000 adults followed for 1 year after starting FM, HbA_1c_ was slightly but significantly reduced (−1 mmol/mol [−0.1%]) after 6 months but had returned to baseline levels at study end [18]. In an even larger nationwide audit on FM use in the UK, HbA_1c_ had dropped by 5.2 mmol/mol (0.5%) after 7.5 months of follow-up [19]. There are also several smaller, short-term, observational studies with no control groups showing lowering of HbA_1c_ after commencement of FM. In a recent meta-analysis, which included data from 21 different studies and 1470 individuals, a mean reduction in HbA_1c_ of −0.55% (−7 mmol/mol) after 2–4 months of FM use was reported [6]. In all these reports, the largest fall in HbA_1c_ was seen in those with higher initial HbA_1c_ levels [6, 17–19]. The results of our study, which by far is the largest observational study hitherto conducted with appropriate IPTW balanced groups, longer-term follow-up and extensive clinical information on both FM users and control individuals, suggest a small but sustained HbA_1c_ improvement with FM use as compared with SMBG. Notably, a lowering of HbA_1c_ during the observation period was also seen in the latter group, which emphasises the importance of a non-FM comparator group. Like in earlier reports, we observed the largest HbA_1c_ reduction in the subgroup of FM users with the highest initial HbA_1c_ levels in the descriptive analyses but an improvement was also seen in the subgroups with intermediate and well-controlled HbA_1c_ and this improvement persisted throughout the observation period. Likewise, in the IPTW analysis the difference in HbA_1c_ between FM users and SMBG controls was sustained.

Whether our observed small IPTW difference in HbA_1c_ between the two groups is clinically significant may be arguable. A non-inferiority margin of 0.3–0.4% in HbA_1c_ is generally considered by the authorities (i.e. European Medicines Agency and US Food and Drug Administration) to be clinically meaningful. As the present difference in HbA_1c_ change between FM users and SMBG controls did not pass that threshold, the clinical relevance is uncertain. It is worth noting that in a recent meta-analysis of randomised controlled trials with more technically advanced CGM devices [20], the estimated weighted mean HbA_1c_ difference compared with SMBG was of comparable magnitude (−0.17% [−2.3 mmol/mol]). The fact that HbA_1c_ on average was lower in our study than in previous reports may have a bearing on the observed modest absolute reduction in HbA_1c_ achieved by FM use. Moreover, it should be noted that we did not have information on the exact time point when FM use was started. As the definition of the index date was the first FM registration in the registry, we cannot rule out the possibility that the registration in some cases took place at the first visit after the initiation of FM. If so, we may have diluted the true effect of FM and consequently under-estimated the actual reduction in HbA_1c_. This notion is supported by the observed drop in HbA_1c_ before index, which was apparent in all HbA_1c_ subgroups. Furthermore, as CGM and FM had been accessible in Sweden to a restricted proportion of adults with type 1 diabetes before the start of registration in the NDR, we cannot exclude the possibility that a small number of individuals in our FM group had started to use FM earlier than the index date, or that some individuals in the control group had used the device temporarily before the observation period. The former scenario may also have contributed to a lower-than-real estimate of the improvement of HbA_1c_ by FM. The latter, however, seems to be of less concern as it has clearly been shown that the effectiveness of CGM rapidly vanishes after its withdrawal [21, 22].

Importantly, we also registered fewer events of severe hypoglycaemia among the FM users than in the SMBG control group, and the risk of experiencing severe hypoglycaemia requiring third-party assistance was reduced by 21%. Similarly, the European, multicentre, randomised controlled IMPACT trial in adults with well-controlled type 1 diabetes showed a substantial (38–46%) reduction in time spent in hypoglycaemia below 3.9 mmol/l in FM users vs control individuals using SMBG, although the trial was not powered to detect any significant difference in the incidence of severe hypoglycaemic events [8, 9]. Our present findings, however, corroborate the results from the Belgium and UK real-world studies, where hospital admissions for severe hypoglycaemia, events of severe hypoglycaemias necessitating third-party assistance and episodes of hypoglycaemic comas were significantly reduced after initiating FM [18, 19]. Taken together, these findings clearly indicate that FM confers a preventive effect on hypoglycaemia exposure, including severe hypoglycaemia, in type 1 diabetes. In high-risk individuals with impaired awareness of hypoglycaemia and recurrent severe hypoglycaemic events, however, use of real-time CGM with hypoglycaemia alerts might be more favourable than FM, as shown by Reddy et al in a short-term randomised, controlled study [23]; using sensor-integrated insulin pump systems with predictive low-glucose insulin-suspend function might be even better by [24].

Our study has several limitations. First, we only had access to HbA_1c_ as a measure of glucose control. It would have been informative to add data on glucose sensor metrics such as time in range and time spent in hyper- and hypoglycaemia but these are not available in the NDR. These metrics are considered to be clinically more informative than HbA_1c_ [25]. Moreover, a simultaneous reduction of time spent in both hypo- and hyperglycaemia, leading to an increase in time in range, may not always be mirrored by a corresponding lowering of HbA_1c_ [8, 9]. Furthermore, we do not have any data on scanning frequency or use of structural education programmes, both of which have been shown to be associated with improvements in glycaemic control with FM [26, 27]. Lastly, we may have residual confounding despite our attempts to create well-balanced study groups. However, all FM users and control individuals were identified from the Swedish NDR in an identical manner. The low SMD values after IPTW indicate that the weighting was successful with low risk of having unbalanced groups.

In Sweden, FM is currently the most widely used glucose sensor device and accounts for nearly 80% of all prescriptions for glucose monitoring systems according to the NDR [5]. This is probably due to the relatively low costs compared with real-time CGM systems [28], and the high patient-reported treatment satisfaction of using the device [18, 29]. To date, there are no studies that have evaluated whether FM (or CGM) systems can improve health outcomes in individuals with type 1 diabetes and the effort to accomplish such a study would take several years. However, the escalating use of glucose sensor-based technologies to facilitate self-management of glucose control in type 1 diabetes will soon make it practically and ethically difficult to perform long-term randomised controlled trials with conventional SMBG as comparator. Instead, a practical alternative may be to perform large well-balanced population-based studies comparing different technologies and outcomes. In this study, we have shown the feasibility of this approach, finding that FM was associated with statistically significant reductions in HbA_1c_ and events of severe hypoglycaemia and that the efficacy of FM was retained over time. However, as the improvement in HbA_1c_ was small in absolute terms, it remains to be assessed whether these benefits will translate into prevention of diabetic microvascular and macrovascular complications, and thus reduced societal costs within the diabetes healthcare systems.

## Supplementary Information

ESM (PDF 276 kb)

## Data Availability

The data that support the findings of this study are not publicly available. The study presented here has been subject to an application to an ethical board and approved for publication related to the specific aim of our research project. With reference to the European General Data Protection Regulation (GDPR), the data are personal data and thereby protected by secrecy.

## Abbreviations

ATE: Average treatment effect for everyone
CGM: Continuous glucose monitoring
CSII: Continuous subcutaneous insulin infusion
FM: Flash glucose monitoring
GBM: Generalised boosted regression model
IPTW: Inverse probability of treatment weighting
MDI: Multiple daily injections
NDR: National Diabetes Registry
SMBG: Self-monitoring of blood glucose
SMD: Standardised mean difference
