# Supplementary material for: Effect of flash glucose monitoring in adults with type 1 diabetes: a nationwide, longitudinal observational study of 14,372 flash users compared with 7691 glucose sensor naive controls
Source: Diabetologia. 2021 Mar 27;64(7):1595–603. doi: 10.1007/s00125-021-05437-z (PMC8187189; doi:10.1007/s00125-021-05437-z)
Supplement: Supplementary file 1 — (PDF 276 kb) [file 125_2021_5437_MOESM1_ESM.pdf]

Supplementary table 1 Crude descriptive statistics, by baseline HbA1c for FM users*

FM users, HbA1c by baseline	≤52 mmol/mol (≤6.9%)	53-69 mmol/mol (7.0%-8.5%)	≥70 mmol/mol (≥8.6%)
**n**	2164	5867	2973
**Age, years (SD)**	44.13 (16.53)	46.73 (16.54)	44.48 (16.76)
**Male sex, N (%)**	948 (43.8)	2684 (45.7)	1347 (45.3)
**Diabetes duration, years (SD)**	21.74 (16.26)	24.94 (15.08)	23.30 (13.97)
**Insulin pump users (CSII), N (%)**	414 (19.1)	1345 (22.9)	581 (19.5)
**Hba1c, mmol/mol (SD)**	46.84 (4.62)	61.08 (4.70)	79.95 (10.52)
**(HbA1c, % NGSP (SD))**	(6.4 (0.4))	(7.7 (0.4))	(9.5 (1.0))
**BMI, kg/m^2 (SD)**	24.91 (3.78)	26.16 (4.16)	26.46 (4.65)
**Systolic BP, mm Hg (SD)**	125.25 (14.18)	126.65 (13.80)	127.64 (15.10)
**Diastolic BP, mm HG (SD)**	73.29 (8.98)	73.66 (9.01)	74.51 (9.48)
**LDL-cholesterol, mmol/L (SD)**	2.42 (0.76)	2.45 (0.77)	2.59 (0.90)
**HDL-cholesterol, mmol/L (SD)**	1.73 (0.50)	1.68 (0.50)	1.56 (0.48)
**Total cholesterol, mmol/L (SD)**	4.56 (0.91)	4.56 (0.92)	4.68 (1.03)
**Triglycerides, mmol/L (SD)**	0.93 (0.62)	1.01 (0.65)	1.23 (0.81)
**Creatinine, µmol/L (SD)**	77.08 (33.31)	77.07 (38.13)	76.49 (34.12)
**eGFR, ml/min/1.73m^2 (SD)**	92.19 (22.98)	91.35 (23.90)	95.07 (28.78)
**Albuminuria, N (%)**			
**No albuminuria**	1751 (91.4)	4476 (86.3)	1981 (78.1)
**Previous albuminuria**	36 (1.9)	165 (3.2)	91 (3.6)
**Microalbuminuria**	89 (4.6)	412 (7.9)	324 (12.8)
**Macro albuminuria**	40 (2.1)	136 (2.6)	140 (5.5)
**Physical activity, N (%)**			
**Never**	71 (3.8)	285 (5.5)	235 (9.2)
**< Once weekly**	185 (9.9)	602 (11.7)	459 (18.0)
**1-2 times weekly**	378 (20.2)	1211 (23.5)	612 (24.0)
**3-5 times weekly**	687 (36.6)	1719 (33.3)	666 (26.1)
**Daily activity**	554 (29.5)	1346 (26.1)	577 (22.6)
**Severe Hypoglycaemia (SH), N (%)**			
**No SH**	1832 (93.0)	5086 (93.9)	2580 (95.3)
**1-2 registered SH**	113 (5.7)	278 (5.1)	105 (3.9)
**3-5 registered SH**	15 (0.8)	28 (0.5)	17 (0.6)
**> 5 registered SH**	10 (0.5)	22 (0.4)	6 (0.2)
**Ischemic heart disease, N (%)**	67 (3.3)	376 (6.8)	218 (7.8)
**Retinopathy, N (%)**	1107 (54.4)	3863 (68.7)	2076 (73.9)
**Stroke, N (%)**	49 (2.4)	154 (2.8)	96 (3.5)
**Smokers, N (%)**	119 (5.9)	471 (8.5)	454 (16.5)

Data are presented as number (proportion, %) for categorical variables or mean (SD) for continuous variables. BMI: Body Mass Index, BP: Blood Pressure, CSII: Continuous Subcutaneous Insulin Infusion, eGFR: estimated Glomerular Filtration rate (calculated with the MDRD formula), HDL: High Density Lipoprotein, LDL: Low Density Lipoprotein, NGSP: National glycohemoglobin standardization program, SH: severe hypoglycaemia (defined as hypoglycaemia requiring assistance from a third party. * Only FM users with non-missing pre index HbA1c included, N=11004.

Supplementary table 2 Crude descriptive statistics, by baseline HbA1c for controls*

**Controls, HbA1c by baseline**	**≤52 mmol/mol (≤6.9%)**	**53-69 mmol/mol (7.0%-8.5%)**	**≥70 mmol/mol (≥8.6%)**
**N**	1383	2879	1121
**Age, years (SD)**	51.83 (19.18)	56.59 (17.43)	55.96 (18.97)
**Male sex, N (%)**	536 (38.8)	1156 (40.2)	508 (45.3)
**Diabetes duration, years (SD)**	21.86 (18.16)	28.08 (16.89)	26.06 (16.28)
**Insulin pump users (CSII), N (%)**	112 (8.1)	268 (9.3)	70 (6.2)
**Hba1c, mmol/mol (SD)**	46.55 (4.65)	60.55 (4.63)	79.79 (10.60)
**(HbA1c, % NGSP (SD))**	(6.4 (0.4))	(7.7 (0.4))	(9.5 (1.0))
**BMI, kg/m^2 (SD)**	25.36 (4.22)	26.22 (4.40)	26.11 (4.69)
**Systolic BP, mm Hg (SD)**	128.43 (14.36)	130.24 (14.90)	130.87 (16.34)
**Diastolic BP, mm HG (SD)**	73.68 (9.12)	72.74 (9.46)	73.02 (10.03)
**LDL-cholesterol, mmol/L (SD)**	2.41 (0.81)	2.44 (0.83)	2.48 (0.88)
**HDL-cholesterol, mmol/L (SD)**	1.68 (0.52)	1.64 (0.51)	1.61 (0.53)
**Total cholesterol, mmol/L (SD)**	4.54 (0.93)	4.54 (0.98)	4.62 (1.05)
**Triglycerides, mmol/L (SD)**	1.02 (0.64)	1.12 (0.74)	1.30 (0.87)
**Creatinine, µmol/L (SD)**	81.68 (48.25)	81.93 (45.16)	82.56 (55.99)
**eGFR, ml/min/1.73m^2 (SD)**	88.20 (24.98)	85.47 (25.26)	88.27 (32.13)
**Albuminuria, N (%)**			
**No albuminuria**	1033 (87.5)	2082 (82.5)	689 (74.1)
**Previous albuminuria**	24 (2.0)	89 (3.5)	25 (2.7)
**Microalbuminuria**	92 (7.8)	258 (10.2)	156 (16.8)
**Macro albuminuria**	31 (2.6)	94 (3.7)	60 (6.5)
**Physical activity, N (%)**			
**Never**	73 (6.3)	222 (9.1)	150 (16.2)
**< Once weekly**	113 (9.8)	323 (13.2)	171 (18.5)
**1-2 times weekly**	215 (18.7)	510 (20.9)	187 (20.2)
**3-5 times weekly**	352 (30.6)	687 (28.1)	194 (21.0)
**Daily activity**	398 (34.6)	699 (28.6)	223 (24.1)
**Severe Hypoglycaemia (SH), N (%)**			
**No SH**	1157 (94.2)	2452 (95.4)	921 (94.2)
**1-2 registered SH**	62 (5.0)	91 (3.5)	47 (4.8)
**3-5 registered SH**	6 (0.5)	17 (0.7)	5 (0.5)
**> 5 registered SH**	3 (0.2)	10 (0.4)	5 (0.5)
**Ischemic heart disease, N (%)**	86 (6.7)	294 (10.8)	143 (14.0)
**Retinopathy, N (%)**	614 (48.8)	1846 (68.4)	756 (74.3)
**Stroke, N (%)**	44 (3.4)	149 (5.5)	79 (7.8)
**Smokers, N (%)**	100 (7.9)	289 (11.0)	199 (19.7)

Data are presented as number (proportion, %) for categorical variables or mean (SD) for continuous variables. BMI: Body Mass Index, BP: Blood Pressure, CSII: Continuous Subcutaneous Insulin Infusion, eGFR: estimated Glomerular Filtration rate (calculated with the MDRD formula), HDL: High Density Lipoprotein, LDL: Low Density Lipoprotein, NGSP: National glycohemoglobin standardization program, SH: severe hypoglycaemia (defined as hypoglycaemia requiring assistance from a third party). * Only controls with non-missing pre index HbA1c included N= 5383.

Supplementary figure 1 General updated mean HbA1c for FM users categorized into low (≤ 52 mmol/mol [≤ 6.9%]), intermediate (53-69 mmol/mol [7.0-8.5%]) and high (≥70 mmol/mol [≥ 8.6%]) HbA1c. a: Male FM users. b: Female FM users. The graphs show unadjusted data and a comparison between the groups based on the figure should therefore be made cautiously as these are not adjusted for confounders.

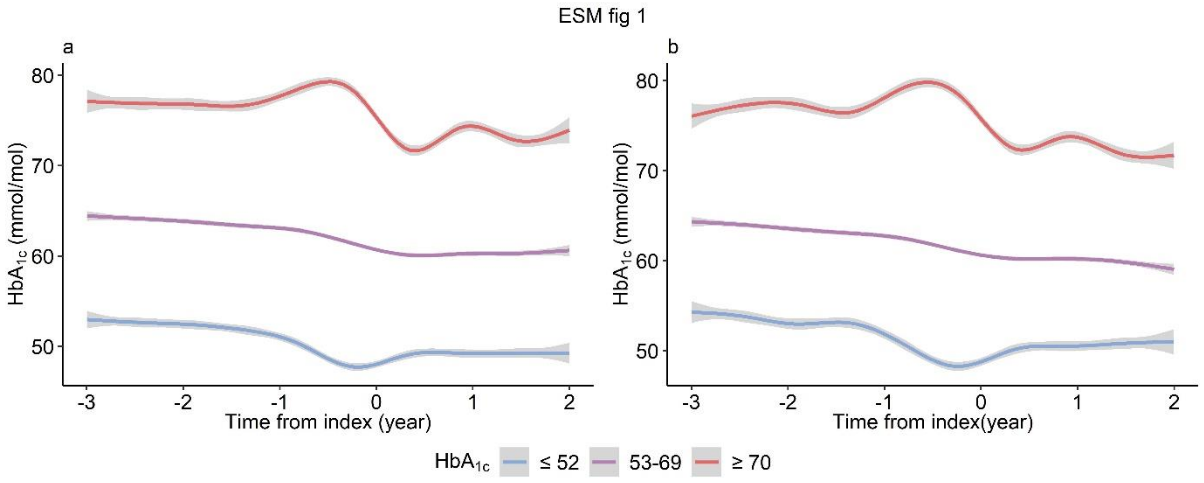

The shaded area depicts indicative confidence intervals for the smooth functions of HbA1c. Note that the confidence interval does not account for multiple repeated measures from the same persons. The spikes in the graph depicting general updated mean for HbA1c observed pre-index in the group with HbA1c ≥ 70 mmol/mol (≥ 8.6%) and the pre-index dips in the group with HbA1c ≤ 52 (≤ 6.9%) mmol/mol are caused by the effect of the "regression to the mean" and are accordingly not associated to an effect of an intervention or selection bias.

FM: Flash glucose monitoring

Supplementary figure 2 General updated mean HbA1c for controls categorized into low (≤ 52 mmol/mol [≤ 6.9%]), intermediate (53-69 mmol/mol [7.0-8.5%]) and high (≥70 mmol/mol [≥ 8.6%]) HbA1c. a: Male controls b: Female controls. The graphs show unadjusted data and a comparison between the groups based on the figure should therefore be made cautiously as these are not adjusted for confounders.

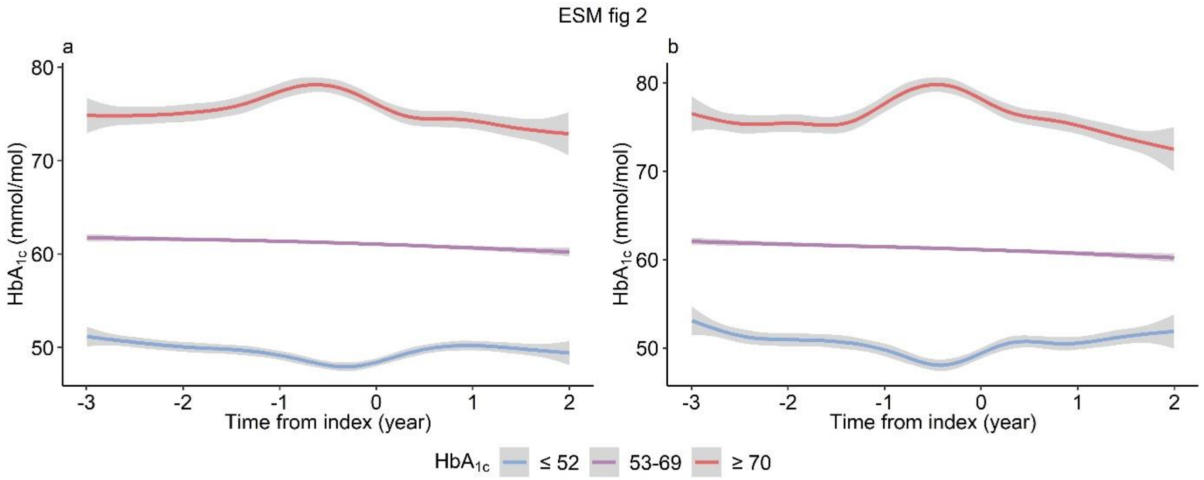

The shaded area depicts indicative confidence intervals for the smooth functions of HbA1c. Note that the confidence interval does not account for multiple repeated measures from the same persons. The spikes in the graph depicting general updated mean for HbA1c observed pre-index in the group with HbA1c ≥ 70 mmol/mol (≥ 8.6%) and the pre-index dips in the group with HbA1c ≤ 52 (≤ 6.9%) are caused by the effect of the "regression to the mean" and are accordingly not associated to an effect of an intervention or selection bias.
